# A comparison of performance of plant miRNA target prediction tools and the characterization of features for genome-wide target prediction

**DOI:** 10.1186/1471-2164-15-348

**Published:** 2014-05-08

**Authors:** Prashant K Srivastava, Taraka Ramji Moturu, Priyanka Pandey, Ian T Baldwin, Shree P Pandey

**Affiliations:** Department of Biological Sciences, Indian Institute of Science Education and Research- Kolkata, Mohanpur Campus, Mohanpur, 741252 West Bengal India; National Institute of Biomedical Genomics, Kalyani, 741251 West Bengal India; Department of Molecular Ecology, Max Planck Institute for Chemical Ecology, Hans-Knoell Str. 8, 07745 Jena, Germany; Integrative Genomics and Medicine, MRC clinical sciences, Imperial College, London, UK

**Keywords:** miRNA, Target prediction, Plants, Deep-sequencing, Non-model plants

## Abstract

**Background:**

Deep-sequencing has enabled the identification of large numbers of miRNAs and siRNAs, making the high-throughput target identification a main limiting factor in defining their function. In plants, several tools have been developed to predict targets, majority of them being trained on Arabidopsis datasets. An extensive and systematic evaluation has not been made for their suitability for predicting targets in species other than Arabidopsis. Nor, these have not been evaluated for their suitability for high-throughput target prediction at genome level.

**Results:**

We evaluated the performance of 11 computational tools in identifying genome-wide targets in Arabidopsis and other plants with procedures that optimized score-cutoffs for estimating targets. Targetfinder was most efficient [89% ‘precision’ (accuracy of prediction), 97% ‘recall’ (sensitivity)] in predicting ‘true-positive’ targets in Arabidopsis miRNA-mRNA interactions. In contrast, only 46% of true positive interactions from non-Arabidopsis species were detected, indicating low ‘recall’ values. Score optimizations increased the ‘recall’ to only 70% (corresponding ‘precision’: 65%) for datasets of true miRNA-mRNA interactions in species other than Arabidopsis. Combining the results of Targetfinder and psRNATarget delivers high true positive coverage, whereas the intersection of psRNATarget and Tapirhybrid outputs deliver highly ‘precise’ predictions. The large number of ‘false negative’ predictions delivered from non-Arabidopsis datasets by all the available tools indicate the diversity in miRNAs-mRNA interaction features between Arabidopsis and other species. A subset of miRNA-mRNA interactions differed significantly for features in seed regions as well as the total number of matches/mismatches.

**Conclusion:**

Although, many plant miRNA target prediction tools may be optimized to predict targets with high specificity in Arabidopsis, such optimized thresholds may not be suitable for many targets in non-Arabidopsis species. More importantly, non-conventional features of miRNA-mRNA interaction may exist in plants indicating alternate mode of miRNA target recognition. Incorporation of these divergent features would enable next-generation of algorithms to better identify target interactions.

**Electronic supplementary material:**

The online version of this article (doi:10.1186/1471-2164-15-348) contains supplementary material, which is available to authorized users.

## Background

Regulatory non-coding small RNAs (smRNAs, 18–30 nucleotides (nt)) play important roles in the regulation of cellular, physiological and ecological processes in plants [1–5]. smRNAs recognize target mRNA molecules by directing effector Argonaute (AGO) protein complexes via base-pairing interactions with nucleic acid molecules, which usually leads to the inhibition of gene expression. In plants, thousands of smRNAs are expressed at any given condition [6, 7]. Elucidation of the function of these smRNAs would largely depend on recognition of their target molecules. Rapid advances in ‘deep-sequencing’ (or ‘next-generation sequencing’; NGS) technology have enabled genome-wide identification of large numbers of smRNAs (including the rare molecules) with greater efficiency [8, 9]. Thus, the current bottleneck in understanding RNA mediated interaction is the correct identification of genes that may be targeted by the numerous smRNAs in the cell.

Plant smRNAs are broadly classified into microRNAs (miRNAs) and small-interfering RNAs (siRNAs). miRNAs are endogenous and originate from specific locations in genomes. The primary transcripts of miRNAs are transcribed in the nucleus in an RNA polymerase II-dependent manner, and transported to the cytoplasm, where these stem-loop structures generate mature miRNAs [10]. siRNAs may have exogenous as well as endogenous origins from viruses, inverted repeats, transposons, transgenes, convergent mRNAs, natural sense-antisense pairs, hairpin RNAs as well as phased siRNAs. Independent of their origin, both the miRNAs and the siRNAs mechanistically depend on the same two families of proteins, the Dicer-like (DCLs) and the AGOs. smRNAs regulate gene expression by binding to the target mRNAs through complementary base-pairing [11].

Three modes of repression of targets have been proposed in plants [12]. First, a large number of plant miRNA targets undergo cleavage [13]. The PIWI domain of the AGO proteins have endonuclease activity that cleave target mRNAs that are complementary to the guide smRNA strand. Plant miRNAs display complementarity to their targets throughout their length and thus help AGOs ‘slice’ targets. This feature of complementarity of miRNA:mRNAs has been (nearly universally) used by the tools that computationally predict miRNA-target interactions in plants [5]. Secondly, translational inhibition of targets, in which the regulation of protein levels occurs without changes in the target’s mRNA levels, has also been suggested in plants [14–17]. Translational repression has generally been associated with the limited complementarity between the miRNAs and the targets in animals. However, the degree of complementarity between the miRNA and mRNA necessary to support translational repression in plants remains unknown [12]. The third mode of action is the ‘destabilization of targets’, in which a minority of plant targets in the degradome do not accumulate slicer cleavage products [18]. Such targets may involve mRNA destabilization instead of slicing. Moreover, the smRNA-target interactions are complex, as one smRNA may regulate the expression of more than one target and one mRNA can be regulated by many smRNAs [14, 19–21]. The role of smRNAs (including miRNAs) in inducible adaptive responses of plants to quick changes in its environment is rapidly being recognized [2, 3, 5, 22, 23]. It is conceivable that such rapid smRNA-mediated adaptive responses may involve mRNA destabilization and the reversible repression of targets in plants.

Although, initial studies in Arabidopsis proposed near-perfect complementarity between the smRNAs and their targets as a general rule, deviations from this rule were soon evident, indicating that pairing at some sites may be less perfect than others [1, 24, 25]. For instance, position 19 of miR319 and position 16 in the target region in the mRNAs were shown to be critical for pairing in Arabidopsis [26]. Similarly, a mismatch at the 10th and 11th positions could lead to inhibition of translation instead of cleavage of target [27]. Furthermore, complementarities at the 3’ end of the miRNA and 5’ end of target has been shown to be more crucial for tasiRNA formation than are the complementarities in the 5’ end of miRNA in Arabidopsis [28]. Such studies indicate that the criteria of perfect/near perfect complementarities between miRNA/mRNAs need to be relaxed and additional features should be included for accurate target prediction. Therefore, features such as the conservation of targets in related species, the location of target sites [in the coding sequence or in the untranslated region (UTR)], cleavage or repression of targets, presence of multiple target sites, target site accessibility, and the integration of expression profiles of both, miRNA and targets have been utilized for predicting targets (as reviewed by [29, 30] and references therein).

Patscan [31] was one of the first tools for predicting targets in Arabidopsis and rice [32] and several new tools have been developed for miRNA target predictions in Arabidopsis. miRU [33], the first tool for the plant-specific miRNA target prediction, which was later upgraded to psRNATarget [34], uses a dynamic programming approach, aligning sequences using a modified Smith-Waterman algorithm and applying the ‘RNAup’ algorithm [35] for target site accessibility. Targetfinder [36] implements a ‘FASTA’ program along with a penalty scoring scheme for mismatches, bulges, or gaps for aligning the sequences. In 2010, two web-servers, TAPIR [37] and Target-align [38], were introduced. TAPIR is imbedded with two search options, the ‘FASTA’ search engine (for ‘fast’ searches), and the ‘RNA hybrid’ search engine (for ‘precise’ results). Target-align also employ the Smith-Waterman based scoring method for predicting the complementarities between miRNAs and mRNAs. Target-align is implemented both as a web server and as a standalone tool, but its utility for genome-wide target predictions for smRNAs has not been tested. Target_Prediction [39] is based on ‘scanning’ targets for miRNA-patterns followed by the calculation of the minimum free energy (with the help of ‘RNAhybrid’) for predicting miRNA-mRNA duplexes. miRTour [40], a web server based program, implements a variety of resources such as BLASTX, RNAfold and ClustalW for the prediction of targets (and thus also involves energy minimizations). imiRTP [41] is an integrated miRNA target interaction prediction tool kit only for *Arabidopsis thaliana* miRNAs. Further, machine learning has been implemented for predicting the plant miRNA targets, for instance, p-TAREF [42] implements support vector regression (SVR) and uses a feature of information of ‘dinucleotide density variation’ around the target site from datasets of *A. thaliana*, *Oryza sativa*, *Medicago truncatula* and *Solanum lycopersicum*. psRobot [43] is a server hosting a toolbox for analyzing plant smRNAs: it has two modules of stem-loop prediction and smRNA target prediction. psRobot uses a modified Smith-Waterman algorithm and target site conservation to predict targets in *A. thaliana, Brachypodium distachyon, Carica papaya, O. sativa, Populus trichocarpa, Sorghum bicolor, Vitis vinifera* and *Zea mays*. psRobot have implemented parallel programming to reduce the run-time during analysis of large datasets such as transcriptomes and genomes.

Although large numbers of tools are available for identifying smRNA-targets *in-silico*, a comprehensive evaluation of these tools for large-scale, genome-wide target identification has been lacking. In the post-genomics era where microarrays and deep-sequencing technologies have enabled unparalleled data production, genome-wide target prediction with high accuracy is becoming critical for the elucidation of functions of smRNAs. Here we have examined the performance of 18 publically available target prediction tools for plants, including the three miRNA-target prediction tools that are extensively used but not explicitly developed for plants (Targetscan [44, 45], miRanda [46], RNAhybrid [47, 48]). We have chosen an experimentally validated dataset from Arabidopsis and other plant species comprising of 330 and 115 unique experimentally validated miRNA:mRNA interactions, respectively, to evaluate the tools.

## Results

**Figure 1:**
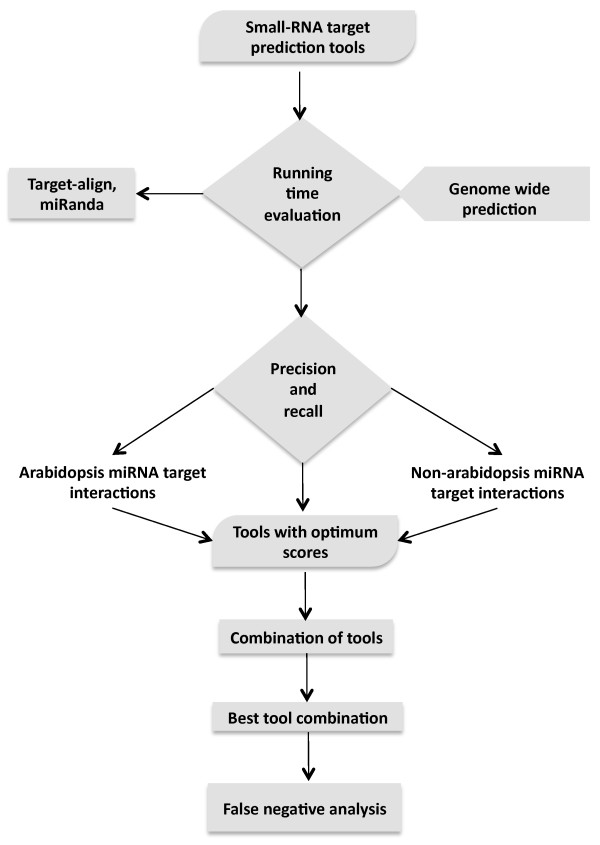
**Schematic representation of the strategy adapted to evaluate smRNA target prediction tool in plants.**

Figure 1 shows an overview of the strategy used to evaluate the performance of plant-specific and other available tools for *in-silico* identification of smRNA-targets and to study the features that may affect miRNA-mRNA interactions. The evaluation and selection of tools was performed in two stages, a) assessing algorithmic efficiency, and b) determining performance of the algorithm on the experimentally validated plant miRNA targets (Figure 1).

### Algorithmic efficiency

While testing the performance of 18 published smRNA target prediction tools (Additional file 1), we found that some of these tools were either discontinued, or their source codes or web servers were not available (for instance miRNAassist [49]). Web miRNA designer, WMD3, [50] is used to custom design artificial miRNAs to silence expression of specific targets, this tool was also not considered fit for genome-wide target prediction, Similarly, the slice detector module of SoMART [51] was not considered because it uses the degradome data for mapping targets of miRNAs. Thus, a total of eleven tools (8 tools specifically published for plant targets and 3 others - Targetscan, miRanda, RNAhybrid) were selected for further evaluation in this study. All the selected tools were implemented either as stand-alone tools, web server or both (Additional file 1). These tools predicted targets for plant miRNAs (from miRBase version 18; [52, 53]), against the *A thaliana’s* trancriptome (Phytozome V8.0; [54]) at their default settings. Initial evaluation of the selected tools was based on a) execution time and b), the average number of targets predicted per miRNA.

**Figure 2:**
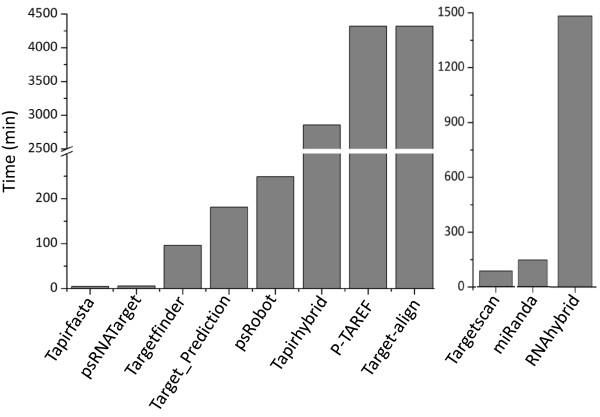
**Computational time required for each of the tools to predict targets in Arabidopsis transcriptome at their default settings.**

Execution time is the time required by a program to predict targets in transcripts for a given set of smRNAs. Execution time for the selected tools varied between 5 minutes to a few weeks (Figure 2). psRobot was the only tool that allowed parallel processing of the dataset. psRNATarget and Tapirfasta were among the fastest, while Target-align and p-TAREF were the slowest tools, each taking <2 weeks to complete target predictions. Due to such long execution times, these tools may not be suitable for high throughput analysis. Computation time for exclusive web server tool (psRNATarget) was not evaluated, since the target prediction may also depend on factors such as load (number of jobs submitted) on the server hosting the tool and/or the internet-speed (Figure 2).

**Figure 3:**
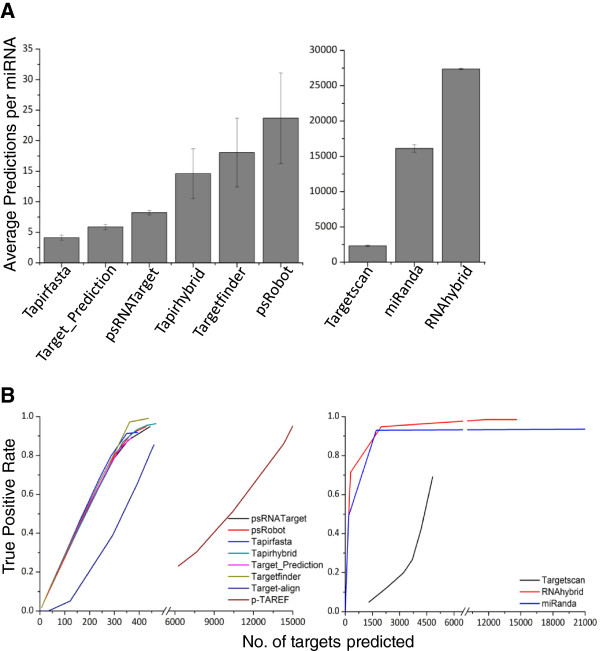
**Genome-wide evaluation of tools for target-prediction in** ***A. thaliana*** **. (A)** Average number of targets predicted by the different tools from the Arabidopsis transcriptome for a miRNA. **(B)** Number of predictions required by different tools for attaining a true positive rate of 1. Error bars represent standard deviations.

The average number of targets predicted by plant specific tools ranged from 5–20 transcripts per miRNA (Figure 3A). This observation is largely consistent with the previous hypothesis, as a single miRNA is capable of targeting multiple (around 6–7) transcripts and a single transcript could be targeted by several (4–5) miRNAs [14, 18]. The majority of the selected tools were trained on the Arabidopsis dataset; therefore these may return the same pattern of target predictions. Indeed, plant specific tools demonstrated a high degree of overlap in target predictions (Additional file 2). Notably, other tools such as miRanda, RNAhybrid and Targetscan, which have been routinely used for target prediction in humans and other model organisms [55], predicted a large number of targets (<4000 transcripts) per miRNA. Although sequence complementarity has been regarded as one of the most critical principles of miRNA target recognition, such high numbers of predictions indicate that these tools use algorithms that may not be relevant to miRNA-targets in plants due to the differences in the mechanisms of target recognition in plants and animals (Figure 3A).

### Performance of the algorithm on experimentally validated plant miRNA targets

*A. thaliana* is one of the best studied plant species including for its smRNAs. For the purpose of this evaluation we broadly classified experimentally validated datasets into two categories, one that originated from Arabidopsis (‘Arabidopsis dataset’) and the other that were obtained from species other than Arabidopsis (‘non-Arabidopsis dataset’).

#### Evaluation of the tools on Arabidopsis dataset

**Figure 4:**
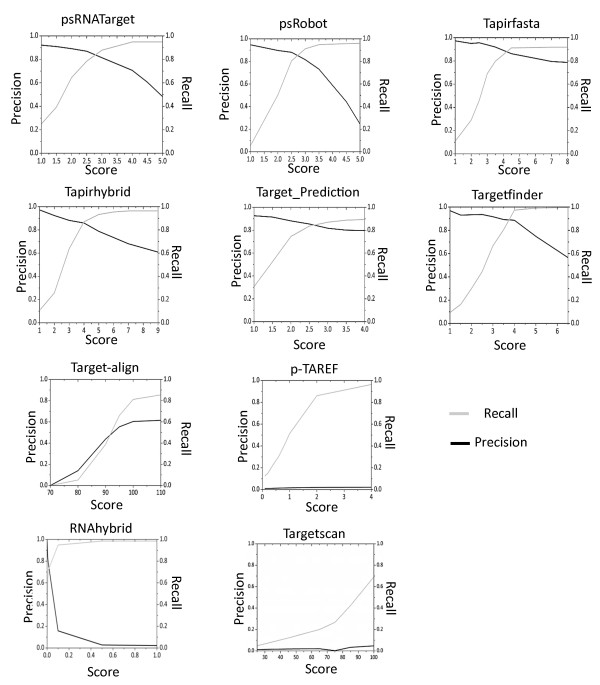
**Comparison of ‘precision’ and ‘recall’ rates for prediction by various tools to determine optimal scores for predictions of targets in Arabidopsis dataset.** The intersection of ‘precision’ and ‘recall’ designates the optimal score for an algorithm.

A plot of the true positive rate (TPR) and the total number of targets predicted (Figure 3B) suggests that a majority of plant specific tools followed a similar distribution: an average of ~400 transcripts was predicted as targets to achieve a TPR of close to one. At this TPR value, the total number of predictions observed for other widely used tools, miRanda, RNAhybrid and Targetscan, exceeded 5000 (Figure 3B). ‘Precision’ and ‘recall’ are important evaluative parameters to measure accuracy and sensitivity of predictions. At their default settings, ‘precision’ of the selected tools were in the range of 0.81 to 0.89 while ‘recall’ ranged between 0.81 and 0.97. To determine the most suitable threshold/cutoffs, ‘precision’ and ‘recall’ were calculated at all possible scores (Figure 4). Scores at which the ‘precision’ and ‘recall’ values intersected were considered to be optimal for the respective tools (Additional file 3). This optimization marginally improved the ‘precision’ and ‘recall’ values for psRobot and Target_Prediction (Additional file 4) in Arabidopsis.

p-TAREF, an algorithm that implements ‘machine learning’ , gave a genome-wide prediction of 15,082 miRNA target interactions with very low ‘precision’ of only 2% (even though it had an increase in sensitivity from 12.7 to 96.3%). Due to extremely low ‘precision’ percentage, this tool was not considered further. In addition, RNAhybrid, miRanda, Target-align and Targetscan were also not further considered as they returned low ‘precision’ and ‘recall’ rates throughout the analysis (Figure 4). Targetfinder performed the best among the selected tools with a ‘recall’ rate of 88% and a ‘precision’ rate of 97% (Figure 4).

**Figure 5:**
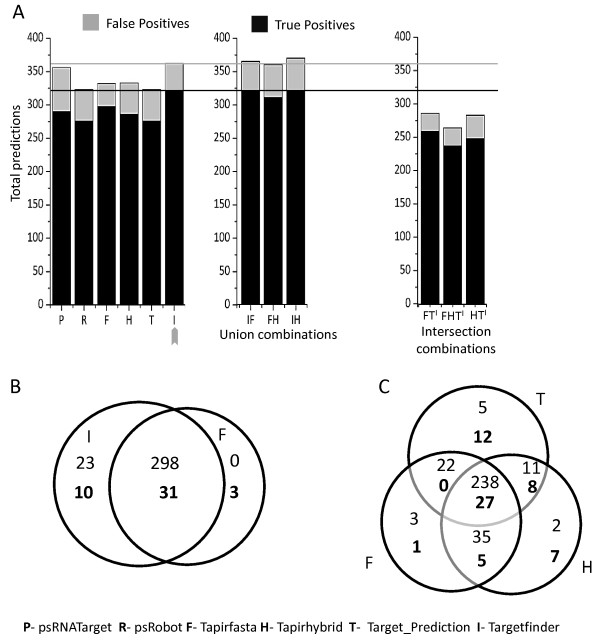
**Combining outputs of individual tools do not affect the performance of predictions in Arabidopsis. (A)** Comparison of true positive and false positive predictions by the top 6 tools for the Arabidospsis dataset. The arrow reveals Targetfinder as returning the maximum number of true positives. Union **(B)** of results (from Targetfinder and Tapirfasta; Tapirfasta results form a subset of results of Targetfinder), or intersection **(C)** of results (from Tapirfasta, Tapirhybrid and Target_Prediction) do not improve prediction rates as compared to those returned by Targetfinder alone. Bold and regular numbers represent false positives and true positives respectively.

Thus, six tools were selected from the initial evaluation of their algorithmic efficiency (run time and genome-wide prediction; Additional file 1), which includes psRNATarget, psRobot, Tapirfasta, Tapirhybrid, Target_Prediction, Targetfinder. Next, results from these six tools were combined with other tools as unions and intersections to improve their ‘precision’ and ‘recall’ (Figure 5). Compared to the outcomes from individual programs, unions of results of two tools could achieve higher recalls. Similarly, the intersections could attain higher precisions. After score optimizations, the combination of tools had only marginal effects on the performance of the tools (Figure 5). Targetfinder performed best (‘precision’ 89%, ‘recall’ 97%) among all the selected tools for the Arabidopsis species at the optimal score of 4.0.

#### Evaluation of the tools on datasets from non-Arabidopsis species

**Figure 6:**
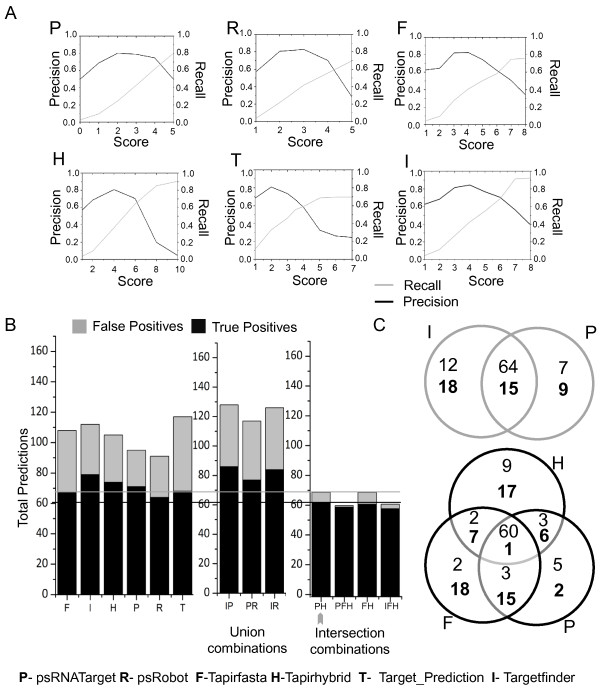
**Evaluation of plant miRNA target prediction tools for identifying true miRNA-mRNA interaction in non-Arabidopsis species. (A)** Comparison of ‘precision’ and ‘recall’ of the six plant specific tools to optimize scores for predicting targets in non-Arabidopsis dataset. **(B)** Comparison of true positive and false positive predictions by 6 tools independently and in-combination. The intersection of psRNATarget and Tapirhybrid (PH, marked with an arrow) delivers the best trade-off between true and false positive rates. Overlap of TP and FN is represented in **(C)** when the union of Targetfinder and psRNATarget (upper Venn diagram) or the intersection of psRNATarget, Tapirhybrid, and Targetfinder (PHI; lower Venn diagram) of predictions are made. Again, bold and regular numbers represent false positives and true positives respectively.

A maximum of 43% ‘recall’ was recorded when tools were scanned against the datasets for species other than Arabidopsis using the ‘optimal scores’ that were obtained earlier for Arabidopsis datasets (Additional file 3). This observation indicates that the optimal cut-off scores for Arabidopsis and non-Arabidopsis datasets may differ and warranted an independent optimization of the tools for the non-Arabidopsis species as well. Optimal scores were calculated for the non-Arabidopsis species in a way similar to those calculated for Arabidopsis (Figure 6 and Additional file 3). Indeed, use of independently evaluated optimal scores for non-Arabidopsis datasets improved the ‘recall’ rates of all the tools in the range of 56 to 69% (Figure 6). Targetfinder (70% ‘precision’ , 69% ‘recall’), psRNATarget (74% ‘precision’ , 62% ‘recall’), and Tapirhybrid (70% ‘precision’ , 64% ‘recall’) were among the best performing tools. In order to further improve the performance of the tools on non-Arabidopsis datasets, different computational approaches/algorithms were combined (Figure 6B and C). We observed that the combination of tools marginally improved the performance of the tools (Figures 6B and C). The union of Targetfinder (I) and psRNATarget (P) increased the prediction of TP by 7 but increased the FP by 9. This would affect the ‘precision’ of the combination at the genome level and may result in prediction of one-third FP. Intersection combinations of tools improved results by increasing ‘precision’ and reducing number of FP. Although it was able to predict only 55% of the positive dataset, the intersection of psRNATarget (P) and Tapirhybrid (H) reduced 17 FP. Thus, the union combination of Targetfinder (I) and psRNATarget (P) may be used for high TP coverage with a greater risk of FP. Similarly, intersection combinations of psRNATarget (P) and Tapirhybrid (H) may be recommended for highly precise predictions.

In addition to the precision and recall analysis we have also performed the ‘receiver operating characteristic (ROC) analysis (Additional file 5) for evaluation of the tools for both, Arabidopsis and non-Arabidopsis datasets. Results of ROC analysis were consistent with the precision and recall analysis. A clear difference in the performance of tools was observed between Arabidopsis and non-Arabidopsis species (Additional file 5). Targetfinder was confirmed as the best performer in both Arabidopsis (area under curve (AUC) = 0.88) as well in non-Arabidopsis species (AUC = 0.78).

### Factors affecting prediction efficiencies

#### Effect of free energy

**Figure 7:**
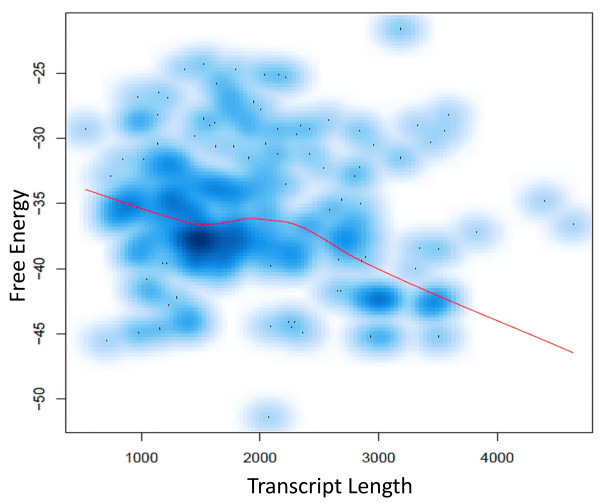
**Relationship between the free energy and the transcript length.** Density plots show how the free energy changes with the increase in length of the mRNAs that were used for the prediction of miRNA-targets. The red line represents the loess based curve fitted data points.

The interaction of miRNA with its target involves the accessibility of targeting site in the mRNA by miRNAs. Such accessibility of mRNA targeting site may be limited by the formation of secondary structures due to folding of the parts of mRNAs at favorable free energies (ΔG). In other words, favorable ΔG condition may govern a true interaction by limiting the accessibility of miRNA binding sites. Interestingly, the majority of target prediction tools incorporate free energy as one of the parameters in their analysis [5, 29, 34, 37, 40, 43]. We computed the free energy to characterize its relationship to transcript length (Figure 7). The free energy values for each of the TP miRNA binding sites in the Arabidopsis dataset is plotted against the length of the respective target. With target length on the x-axis and its respective free energy on the y-axis, a density plot was constructed. Loess curve fitting is a commonly used, non-parametric based technique that fits a smooth curve to the empirical data used for the data points. Density plot and loess based curve fitting was done in R v2.12. We observed that longer transcripts tend to have less free energy (Figure 7). Our observation suggests that depending on the length of the input mRNA sequence the free energy changes. It is important to note here that none of the current tools provide any recommendations on the length of mRNA for the input; this could be potentially another source from where bias in prediction could be introduced.

#### Features of the false negative predictions

**Figure 8:**
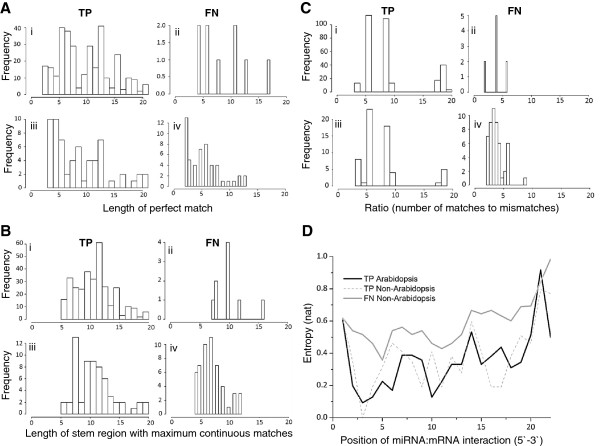
**Characterization of features of true positive (TP) and false negative (FN) predictions in Arabidopsis and non-Arabidopsis datasets. (A)** Distribution of first stretch of miRNA-mRNA targets in TPs and FNs for Arabidopsis and non-Arabidopsis datasets: i) and ii) show the length distribution for the TP and FN miRNAs in Arabidopsis dataset, respectively, while iii) and iv) show the length distributions for TP and FN datasets in non-Arabidopsis dataset, respectively. **(B)** Comparison of the ‘seed region’ (miRNA region with maximum continuous matches with its targets) in TPs and FNs for Arabidopsis and non-Arabidopsis dataset: i) and ii) are the lengths of seed region distributions for the TP and FN miRNAs in Arabidopsis species, respectively, while iii) and iv) represent the lengths of seed region distributions for TP and FN datasets in non-Arabidopsis datasets respectively. **(C)** Distribution of the match-mismatch ratio (number of matches divided by the number of mismatches for miRNA with its target sequences) in TPs and FNs for Arabidopsis and non-Arabidopsis datasets. i) and ii) show the match-mismatch ratio for the TP and FN miRNAs in Arabidopsis dataset respectively, while iii) and iv) are match-mismatch ratios of seed region distributions for TP and FN datasets in non-Arabidopsis dataset respectively. **(D)** Change in entropy with respect to the position of the miRNA:mRNA interaction. Entropy value for each miRNA-mRNA positions for TP predictions in Arabidopsis and non-Arabidopsis datasets and FN predictions for non-Arabidopsis datasets are plotted in black, red and blue respectively.

All the available tools predicted very high numbers of FN when they were tested on non-Arabidopsis datasets (Figure 6B). To characterize the differences in the TP rates observed for the Arabidopsis and non-Arabidopsis datasets, four features were determined, namely (a) the GC content of the miRNAs (Additional file 6), (b) the length of the miRNA seed region (Figure 8A), (c) the first stretch of the stem region (Figure 8B), and (d) the ratio of the number matches and the number of mismatches (Figure 8C) for miRNA-mRNA targets in TP and FN for Arabidopsis and non-Arabidopsis species. GC content of miRNA plays an important role in determining the putative targets [56], however, this feature was not observed to be significantly different across the datasets. In plants, it has been hypothesized that the miRNA-mRNA complexes are near perfect matches [30]. So, we have defined a new metric, maximum matched region as a part of the miRNA-target region with maximum number of continuous matches. We found that this feature differs between the TP and FN of the non-Arabidopsis species (Wilcoxon rank test p-value = 1.7e-05, Figure 8A). This feature did not show a significant difference between the TPs of Arabidopsis and non-Arabidopsis species (Wilcoxon rank test P-value < 0.01). This indicates that a sub-set of non-Arabidopsis miRNAs may differ from Arabidopsis miRNAs in the way they interact with their targets. A variant of this feature could be to characterize the first stretch of the miRNA region that matches perfectly with its target sequences until a mismatched base is observed. Such a mechanism would be more consistent with the conventional definition of the seed region [57]. When this idea was tested, a similar trend of significant differences between the TP and FN datasets of the non-Arabidopsis species (Wilcoxon rank test P-value = 1.4e-11; Figure 8B) but not between the TP datasets of Arabidopsis and non-Arabidopsis species (Wilcoxon rank test P-value < 0.01; Figure 8B) was observed. In order to assess if the differences in the continuous matching region is also affected by the number of mismatches between the miRNA and the mRNA target sequences, we calculated the ratio of the total number of matches over total number of mismatches. Interestingly, the result was consistent as with the previous analysis i.e. no significant differences were observed for the TPs of the Arabidopsis and non-Arabidopsis species (Wilcoxon rank test P-value < 0.01; Figure 8C) while returning significant differences between the TPs and FNs of the non-Arabidopsis species (Wilcoxon rank test P-value = 2.4e-13; Figure 8C).

The differences between the TP and FN at the alignment level are summarized as a heatmap (Additional file 7). The heatmap clustering is based on the alignment of first 20 nt of miRNA-mRNA complementarity of the respective interactions that were plotted with respect to miRNA 5’-3’ direction (Additional file 7). miRNA-mRNA seed interaction positions (2–12 position) can be represented either as (i) less than two G:U wobbles, (ii) two mismatches and one G:U wobble, (iii) only three mismatches and no G:U wobble, with or without mismatch/gap/G:U wobble at the 3’ end of the miRNA target interaction. This indicates that continuous pairing of miRNAs and mRNAs in TP dataset is different from FN datasets in non-Arabidopsis species. This observation is further confirmed by evaluating the entropy values for each miRNA-mRNA positions in TP and FN datasets (Figure 8D). Since, the numbers of FNs in Arabidopsis dataset were too few to deduce any meaningful statistics, we did not use Arabidopsis FNs dataset for further comparisons. Wilcoxon rank-test between TP datasets (Arabidopsis and non-Arabidopsis) were not observed to be significant while Wilcoxon rank test between non-Arabidopsis FN and TP datasets were observed to be highly significant (Wilcoxon rank test P = 4.8e-07 for Arabidopsis and for non-Arabidopsis datasets). This suggests that the entropy values in FN datasets are significantly higher when compared to the TP datasets (Arabidopsis and non-Arabidopsis).

## Discussion

The integration of smRNAs into the existing functional genomics datasets is essential for a better understanding of the cellular, physiological and ecological processes [1, 4, 5, 7]. In the post-genomics era where information on identity, sequence and expression of smRNA is readily attainable, the high-throughput characterization of their targets is proving to be the limiting factor in understanding smRNA-functions. Although several models and computational tools have been proposed for *in-silico* identification of smRNA targets, predicting targets with significant statistical confidence in high-throughput experiments still remains a challenging task. Successful prediction of targets depend on the fine-tuning of several factors effecting the miRNA-mRNA complex formation, such as complementarity of smRNA and target sequence, continuous stretches of matches and mismatches, local structural properties, target site accessibility, free energy of interaction, GC content, G:U pairs, etc. Previously proposed models/tools have been theoretically shown to have optimal performance but a critical comparative evaluation of these algorithms based on experimentally validated dataset has not been performed. In this study we attempted to compare all the available tools for high-throughput smRNA target prediction in Arabidopsis as well as non-Arabidopsis species at optimized scores. In addition, we have tried to understand the possible features that significantly reduce the ‘precision’ and ‘recall’ of tools when they are used for non-Arabidopsis species.

The initial evaluation of the selected eleven plant and animal specific tools were based on the true positive datasets obtained for the *A. thaliana* species (Figure 1). Based on the execution time and the number of FP target predictions, p-TAREF, RNAhybrid, miRanda, Target-align and Targetscan were not considered suitable for genome-wide prediction in plants and therefore these tools were not evaluated further (Figures 2 and 3).

In comparison to their default settings, the optimization of cut-off scores led to an increase in the ‘precision’ of Target_Prediction by 2% (Additional file 4, Figure 4), and to an increase in ‘recall’ by 3% for psRobot. After score optimization, Targetfinder was found to be the most useful tool for predicting targets for Arabidopsis datasets (Figure 5).

We carried out the same set of evaluations for interactions known in non-Arabidopsis species. A maximum of 43% ‘recall’ rate could be obtained when the tools used Arabidopsis ‘optimal score’. By recalculating the cut-off scores for non-Arabidopsis datasets independently, the ‘recall’ rates were increased to the range from 56-69% (Figure 6 and Additional file 4). For the non-Arabidopsis species, we observed that psRNATarget and Tapirhybrid displayed the best trade-off between ‘precision’ and ‘recall’ rates. Depending upon the context of usage (i.e. rapid sensitive scans or highly specific predictions) a combination of union or intersection for these tools could be recommended for miRNA target prediction in non-Arabidopsis species (see Results for details). This observation of different ‘optimal score’ and low ‘recall’ rate might suggest an alternate mechanism of target identification for the smRNAs in non-Arabidopsis species.

It has been shown earlier that in the absence of ‘real’ TN dataset precision and recall might prove to be a better measure for the tool evaluation [58–61]. Indeed, a ‘real’ TN dataset for miRNA-mRNA interaction has largely been missing. Still, we have performed ROC analysis with the negative dataset for miRNAs downloaded from a previously published study [42]. ROC analysis clearly suggested that there was a stark difference in the performance of tools between Arabidopsis and non-Arabidopsis species, which is consistent with the precision and recall analysis (Additional file 5, Figures 4 and 6).

We investigated the possible reason for differences in the TP rates observed for the Arabidopsis and non-Arabidopsis species by considering (a) commonality in the prediction programs, and (b) characterizing features of interactions for the miRNAs that were falsely predicted to be negative. Several prediction programs use the hypothesis that low free energy is required for the formation of stable RNA-RNA duplex [33–35, 37, 40, 43, 47]. Due to the limited numbers of solved secondary structures of RNA duplexes, calculations of free energy mainly rely on modeling efforts [62]. We observed that there was a negative association of free-energy with transcript length (Figure 7). This association could introduce bias in the analysis and indicates that universal cut-off scores might not work for all the transcripts of different lengths.

To characterize the attributes for the targets that were falsely predicted to be negative, four features for miRNA-mRNA targets in TPs and FN for Arabidopsis and non-Arabidopsis species were determined (Additional file 6, Figure 8A-C). With an exception of miRNA GC content, we observed that all the other features were significantly different between TP and FNs of the non-Arabidopsis species while no significant differences were observed between TP of Arabidopsis and non-Arabidopsis species. These observations, in addition to the relatively high values for the entropy (Figure 8D) in the FN datasets further suggest that for a sub-set of the miRNAs belonging to the non-Arabidopsis species, additional components of mechanism of the target recognition are likely to exist.

## Conclusions

In this study we have evaluated several miRNA target prediction tools. We observed that the majority of the plant specific tools may be made to predict targets with a high specificity in the model organism, *A. thaliana* if the parameters of predictions are optimized. We further conclude that such optimized ‘scores’ of Arabidopsis may not be used as a threshold while analyzing non-model organism (i.e. non-Arabidopsis datasets); in addition, we have optimized the scores for non-Arabidopsis species. Based on our results of the evaluation of known interactions, Targetfinder alone or in combination with psRNATarget or Tapirhybrid for the miRNA target predictions provided the most satisfactory results. While analyzing the FN datasets, we noted that additional features of target recognition likely exists, which indicates towards possible novel modes of miRNA-mRNA target recognition in non-Arabidopsis plants.

## Methods

### SmRNA target prediction tools

A total of 18 tools were found published but only 11 tools were quantitatively available for sequential evaluation based on different criteria (Additional file 1). Plant specific smRNA target prediction tools that are implemented either in the form of a web server or as a stand-alone tool were included in this study. A summary of all the selected tools is presented in Table 1. Both the source-codes and the web servers are publically available for TAPIR version 1.1, Target-align (Windows version), psRobot version 1.2 and p-TAREF (Linux version), while for Targetfinder release 1.6, Target_Prediction and psRNATarget, either only the source codes or web servers are available. In addition, some of the widely used tools that are not plant specific, such as miRanda (August 2006) [46], RNAhybrid version 2.1 [47, 48] and Targetscan version 6.2 (non-conserved) [44, 45] were also included in our study. The Tapirfasta and Tapir RNAhybrid (Tapirhybrid) search engines [37] were independently evaluated.

**Table 1:** **Comparison of parameters used in the different miRNA target prediction algorithms**

Tool/Parameters	Algorithm	Seed pairing	Target site accessibility	Multiple sites	Conservation filter	Expression profile	Translation inhibition	Availability
Targetfinder	FASTA	+	-	-	-	-	-	Only source code
TAPIR	FASTA/RNAhybrid	+	+	+	-	-	-	Web server and source code
Target-align	Smith-Waterman like	-	-	+	-	-	-	Web server and source code
Target_Prediciton	Scan for matches and RNA hybrid	-	+	-	-	-	-	Only source code
psRNATarget	Smith-Waterman	-	+	+	-	-	+	Only web server
p-TAREF	Support Vector Regression (SVR)	-	+	+	-	+	-	Web server and source code
psRobot	Modified Smith-Waterman	-	-	+	+	+	-	Web server and source code
miRanda	Local Alignment	+	+	+	-	-	+	Web server and source code
RNAhybrid	Intramolecular hybridization	+	+	+	-	-	+	Web server and source code
Targetscan 6.2 (Non- conserved)	Custom made	+	-	+	-	-	+	Only source code

**‘+’** Represent feature used, ‘**-‘** indicates that these features were not used.

### SmRNA-target datasets and genome-wide target prediction

*A. thaliana* is a widely used model flowering plant, for which, the majority of the tools have been developed. As expected, most miRNA-mRNA interactions have also been experimentally validated in *A. thaliana*. These experimentally validated interactions of miRNAs and mRNAs in *A. thaliana* were obtained from the Arabidopsis small RNA project (ASRP) database [63, 64], and by performing a literature search [3, 14, 36, 65]. A majority of these miRNAs were conserved in other species as reported in miRBase V18 [52, 53]. miRNA targets for non-conserved miRNAs (those not reported in *A. thaliana*) included interactions from *O. sativa, Glycine max* and *V. vinifera*; these were curated from the literature [66–71]. Non-redundant miRNA-mRNA interactions add up to 330 for Arabidopsis and 134 for the other plant species (Additional file 8). We have removed miRNAs miR414 and miR413 since they are marked as ambiguous in miRBase; the total number of interactions for other plant species is now 115 (Additional file 8). Thus, our study provides a useful resource by curating a list of experimentally validated miRNA-mRNA interactions in Arabidopsis as well as those only in species other than Arabidopsis. Mature miRNA sequences and target sequences for the Arabidopsis dataset were extracted from miRBase V18 [52, 53] and Phytozome V8.0 [54]. We then evaluated how various computational tools performed in predicting miRNA targets when subjected to large-scale datasets (at genome scales) from Phytozome V8.0 [54]. These datasets were used as inputs to various tools (at their default parameters) for *in-silico* miRNA target identification. The following metrics were evaluated: 1) execution time, and 2) the average of number of targets predicted per miRNA. Ubuntu version 12.04 (64 bit) was used as an operating system for the evaluation of selected tools on Intel® Core™ i7-2600 CPU with a clock rate of 3.40GHz and 16 GB RAM.

### Performance evaluation

Performance of the tools was evaluated by estimating the parameters of ‘precision’ (TP/(TP + FP)) and ‘recall’ (TP/(TP + FN)) [72, 73]. True positives (TP) were defined as experimentally validated *in-silico* predictions; false negatives (FN) are the experimentally validated interactions but not predicted *in-silico*, and the false positives (FP) are defined as *in-silico* predictions that were not experimentally validated. Maximum TPR values were obtained at the following threshold: psRNATarget: 5 (Maximum expectation), Tapirfasta: 10 (Score), Tapirhybrid: 10 (Score), psRobot: 5 (Penalty score threshold), Target_Prediction: 4 (Penalty score), Targetfinder: 6.5 (Prediction Score), Target-align: 110 (Maximum Score), p-TAREF: 4 (SVR score), RNAhybrid: 1 (p-value). ‘Precision’ and ‘recall’ are inversely related to each other: choosing a score cut-off based on a high ‘precision’ value would provide accurate results with low sensitivity and vice-versa. Therefore, an optimal balance between these two parameters is required. In this study, we have defined an optimal score where the ‘precision’ and ‘recall’ values intersect.

ROC analysis was performed by estimating the parameters of ‘specificity’ (TN/(TN + FP)) and ‘sensitivity’ (TP/(TP + FN)) [72, 73]. True positives (TP) were defined as experimentally validated *in-silico* predictions; false negatives (FN) are the experimentally validated interactions but not predicted *in-silico*, and the false positives (FP) are defined as *in-silico* predictions that were not experimentally validated; true negative (TN) dataset (119 sequences) was obtained from a previous publication [42].

### Combinations of prediction algorithms

To further improve the ‘precision’ and ‘recall’ rates, predictions obtained from the selected tools at their optimal scores were combined. Combinations of the tools were assessed by either taking a union or an intersection of the predictions.

### Complementarity and free energy

The uncertainty in the alignment of miRNA:mRNA interaction was measured using Shannon entropy. The observed entropy value (Hn) at each position of the experimentally validated miRNA:mRNA interaction pairs is obtained from the formula:

The experimentally validated dataset from Arabidopsis were used for the free energy calculations using the RNAhybrid free energy tool [47, 48].

### Evaluation of the ‘FN’ dataset

Four features were evaluated to characterize FN predictions. (a) The GC content of miRNAs (percentage of GC in the miRNA sequence) was calculated for the TP and FN datasets in both Arabidopsis and non-Arabidopsis datasets, (b) the length of the miRNA seed region, in which, the miRNA seed region was defined as the longest stretch of complementary bases between miRNA and mRNA, (c) the first stretch of the stem region, where this feature was defined as length of the continuous matches from first matched base until a mismatched base was observed, and (d) the ratio of the total number matches and the total number of mismatches. Differences in alignments are summarized as a heatmap. The heatmaps are based on the alignment between TP miRNA-mRNA pairs using ‘fasta’ sequence alignment tool version 35 [74]. It is possible that different miRNAs may have different lengths. To cope with the variability in the alignment lengths, we have considered the first 20 nt for clustering. Match, mismatch/gap and G:U wobble were given a score +1, −1, and 0.5 respectively. Clustering is based on Euclidean distance and dendrogram was computed using the complete clustering method. All analyses were performed using R 2.15.2 [75].

## Electronic supplementary material

Additional file 1: **Workflow for the selection of miRNA-target prediction tools.** (PDF 269 KB)

Additional file 2: **Pair-wise comparisons of the predictions made by the selected tools.** Total number of predictions obtained from all the selected tools is presented on the diagonal of the matrix and their corresponding overlap with other tools is presented in the subsequent columns. (PDF 95 KB)

Additional file 3: **Optimal cut-off scores for the tools in Arabidopsis and non-Arabidopsis datasets.** (DOCX 15 KB)

Additional file 4: **Comparison of ‘precision’ and ‘recall’ parameters at default and optimized scores for different tools in Arabidopsis and non-Arabidopsis datasets.** (DOCX 16 KB)

Additional file 5: **ROC plots to compare the sensitivity and specificity of the predictions made by various tools in (A) Arabidopsis and (B) Non-Arabidopsis species.** (C) Area under curve (AUC) is tabulated for both Arabidopsis and Non-Arabidopsis species. (PDF 252 KB)

Additional file 6: **Comparison of the GC content distributions for miRNA targets in TPs and FN for Arabidopsis and non-Arabidopsis datasets.** (A) and (B) show GC content distribution for the TP and FN miRNAs in Arabidopsis dataset respectively, while (C) and (D) are the plots of GC content distributions for TP and FN datasets in non-Arabidopsis dataset respectively. (PDF 99 KB)

Additional file 7: **Heatmaps representing the TP (A and B) and FN (C and D) miRNA-mRNA interactions in Arabidopsis and non-Arabidopsis datasets respectively.** (PDF 165 KB)

Additional file 8: **List of miRNA-mRNA interactions that are reported in Arabidopsis and conserved in other species (Arabidopsis dataset; A-C) and those reported from other species but not conserved in Arabidopsis (non-Arabidopsis dataset; E-G).** (XLS 54 KB)
