# Supplementary material for: A comparison of performance of plant miRNA target prediction tools and the characterization of features for genome-wide target prediction
Source: BMC Genomics. 2014 May 8;15(1):348. doi: 10.1186/1471-2164-15-348 (PMC4035075; doi:10.1186/1471-2164-15-348)
Supplement: Supplementary file 1 — Additional file 1: Workflow for the selection of miRNA-target prediction tools. (PDF 269 KB) [file 12864_2014_6052_MOESM1_ESM.pdf]

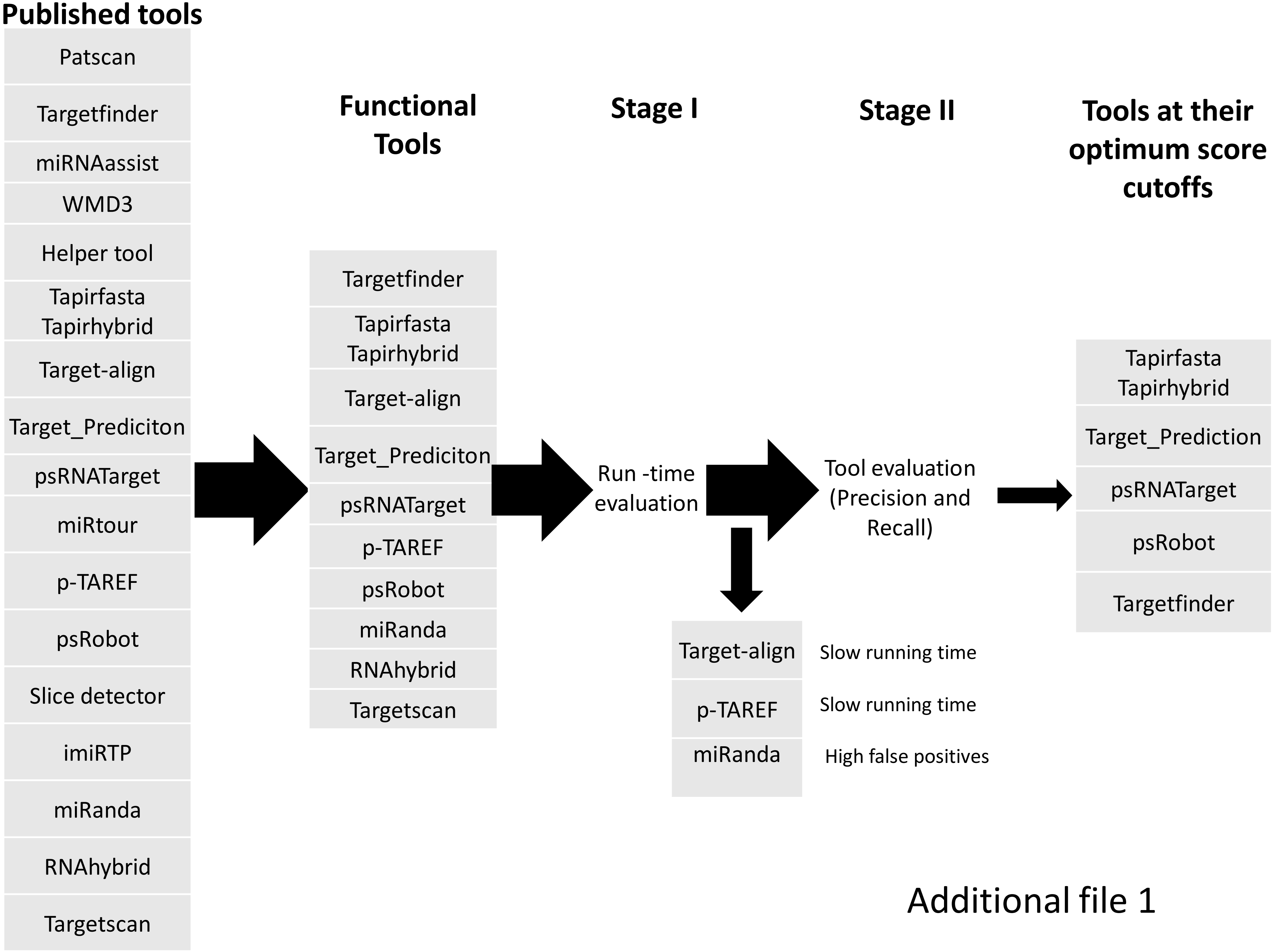

Additional file 1
