# Supplementary material for: A comparison of performance of plant miRNA target prediction tools and the characterization of features for genome-wide target prediction
Source: BMC Genomics. 2014 May 8;15(1):348. doi: 10.1186/1471-2164-15-348 (PMC4035075; doi:10.1186/1471-2164-15-348)
Supplement: Supplementary file 2 — Additional file 2: Pair-wise comparisons of the predictions made by the selected tools. Total number of predictions obtained from all the selected tools is presented on the diagonal of the matrix and their corresponding overlap with other tools is presented in the subsequent columns. (PDF 95 KB) [file 12864_2014_6052_MOESM2_ESM.pdf]

	miRanda	psRNATarget	psRobot	RNAhybrid	Tapirfasta	Tapirhybrid	Targetfinder	Target_Prediction	Targetscan
miRanda	5501298	2614	7121	365567	1377	4517	5570	1944	754848
psRNATarget		2789	1916	1794	1095	1508	1543	1144	1140
psRobot			8004	4383	1256	3273	4340	1488	4087
RNAhybrid				9282494	939	3394	3027	1235	105484
Tapirfasta					1420	1304	1325	733	1322
Tapirhybrid						4986	3747	1083	3310
Targetfinder							6147	1184	3885
Target_Prediction								2027	944
Targetscan									782212

Additional file 2
