# Supplementary material for: A comparison of performance of plant miRNA target prediction tools and the characterization of features for genome-wide target prediction
Source: BMC Genomics. 2014 May 8;15(1):348. doi: 10.1186/1471-2164-15-348 (PMC4035075; doi:10.1186/1471-2164-15-348)
Supplement: Supplementary file 3 — Additional file 3: Optimal cut-off scores for the tools in Arabidopsis and non-Arabidopsis datasets. (DOCX 15 KB) [file 12864_2014_6052_MOESM3_ESM.docx]

Additional file 3: Optimal cut-off scores for the tools in Arabidopsis and non-Arabidopsis datasets.

| Tool | Arabidopsis | Non-Arabidopsis | |
| --- | --- | --- | --- |
|  | Score | Score | Free energy ratio |
| psRNATarget | 3.0 | 4.0 | 50.9* |
| psRobot | 2.8 | 4.0 | - |
| Tapirfasta | 4.0 | 6.0 | 0.55 |
| Tapirhybrid | 4.0 | 6.0 | 0.52 |
| Target_Prediction | 2.5 | 4.0 | 0.54 |
| Targetfinder | 4.0 | 6.0 | - |

*UPE: Maximum energy to unpair the target site
