# Supplementary material for: A comparison of performance of plant miRNA target prediction tools and the characterization of features for genome-wide target prediction
Source: BMC Genomics. 2014 May 8;15(1):348. doi: 10.1186/1471-2164-15-348 (PMC4035075; doi:10.1186/1471-2164-15-348)
Supplement: Supplementary file 4 — Additional file 4: Comparison of ‘precision’ and ‘recall’ parameters at default and optimized scores for different tools in Arabidopsis and non-Arabidopsis datasets. (DOCX 16 KB) [file 12864_2014_6052_MOESM4_ESM.docx]

Additional file 4: Comparison of ‘precision’ and ‘recall’ parameters at default and optimized scores for different tools in Arabidopsis and non-Arabidopsis datasets.

| Tool | Arabidopsis | | | | | | | | Non-Arabidopsis | | | | | | | |
| --- | --- | --- | --- | --- | --- | --- | --- | --- | --- | --- | --- | --- | --- | --- | --- | --- |
|  | Default | | | | Optimized | | | | Default | | | | Optimized | | | |
|  | S | E | P | R | S | E | P | R | S | E | P | R | S | E | P | R |
| psRNATarget | 3 | 25 | 0.81 | 0.89 | 3 | 25 | 0.81 | 0.89 | 3 | 25 | 0.76 | 0.32 | 4 | 50.9 | 0.74 | 0.62 |
| psRobot | 2.5 | - | 0.88 | 0.81 | 2.8 | - | 0.87 | 0.84 | 2.5 | - | 0.81 | 0.30 | 4 | - | 0.70 | 0.56 |
| Tapirfasta | 4 | 0.7 | 0.89 | 0.9 | 4 | 0.7 | 0.89 | 0.9 | 4 | 0.7 | 0.82 | 0.39 | 6 | 0.55 | 0.62 | 0.58 |
| Tapirhybrid | 4 | 0.7 | 0.86 | 0.87 | 4 | 0.7 | 0.86 | 0.87 | 4 | 0.7 | 0.82 | 0.37 | 6 | 0.52 | 0.70 | 0.64 |
| Target_Prediction | 4 | 0.73 | 0.83 | 0.88 | 2.5 | 0.73 | 0.85 | 0.84 | 4 | 0.73 | 0.71 | 0.41 | 4 | 0.54 | 0.58 | 0.59 |
| Targetfinder | 4 | - | 0.89 | 0.97 | 4 | - | 0.89 | 0.97 | 4 | - | 0.84 | 0.43 | 6 | - | 0.70 | 0.69 |

*S Score; E Free energy; P Precision; R Recall
