# Supplementary material for: A comparison of performance of plant miRNA target prediction tools and the characterization of features for genome-wide target prediction
Source: BMC Genomics. 2014 May 8;15(1):348. doi: 10.1186/1471-2164-15-348 (PMC4035075; doi:10.1186/1471-2164-15-348)
Supplement: Supplementary file 5 — Additional file 5: ROC plots to compare the sensitivity and specificity of the predictions made by various tools in (A) Arabidopsis and (B) Non-Arabidopsis species. (C) Area under curve (AUC) is tabulated for both Arabidopsis and Non-Arabidopsis species. (PDF 252 KB) [file 12864_2014_6052_MOESM5_ESM.pdf]

A

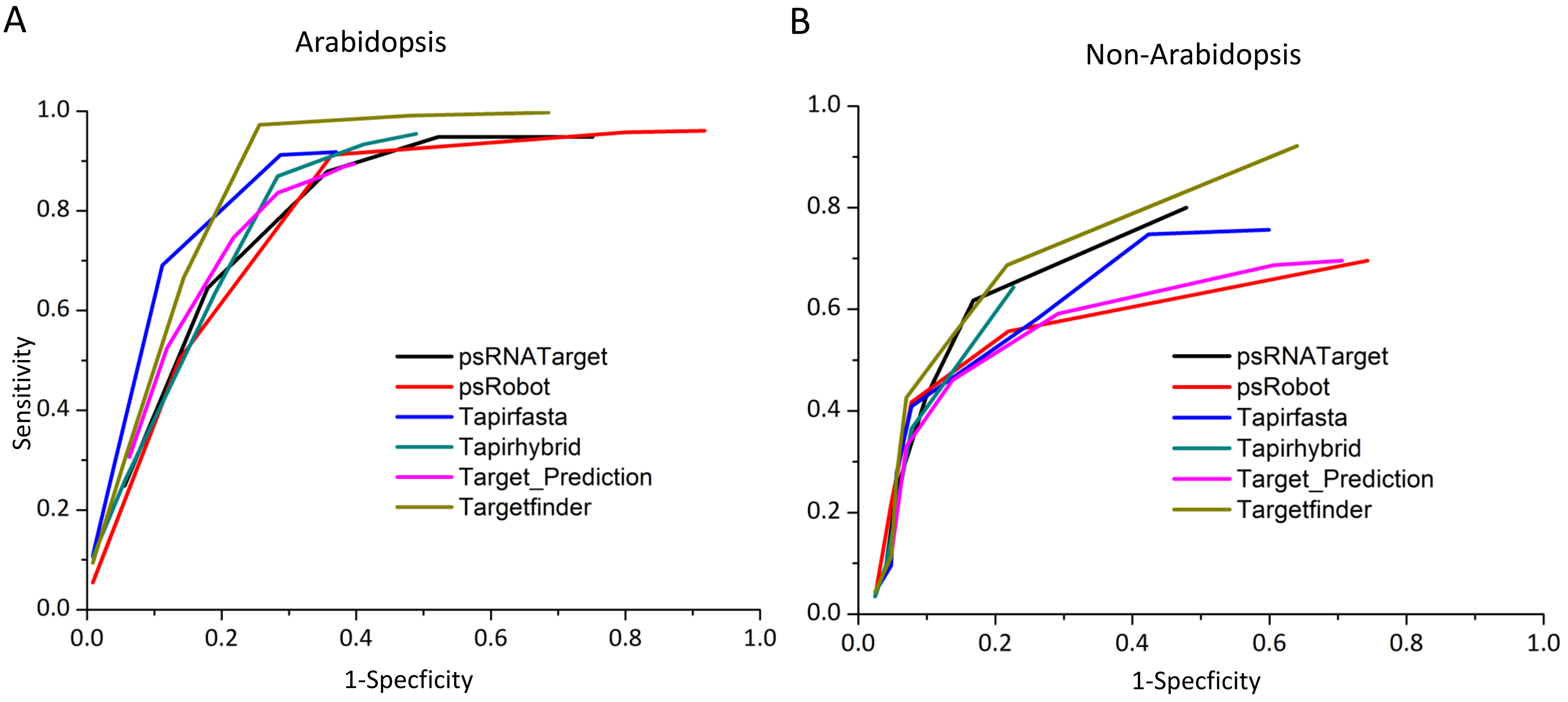

C

Tools/ Area Under Curve (AUC)	psRNATarget	psRobot	Tapirfasta	Tapirhybrid	Target_Prediction	Targetfinder
Arabidopsis	0.81	0.8	0.86	0.83	0.82	0.88
Non-Arabidopsis	0.74	0.63	0.69	0.72	0.63	0.78
