# Supplementary figures and images for: A comparison of performance of plant miRNA target prediction tools and the characterization of features for genome-wide target prediction
Source: BMC Genomics. 2014 May 8;15(1):348. doi: 10.1186/1471-2164-15-348 (PMC4035075; doi:10.1186/1471-2164-15-348)

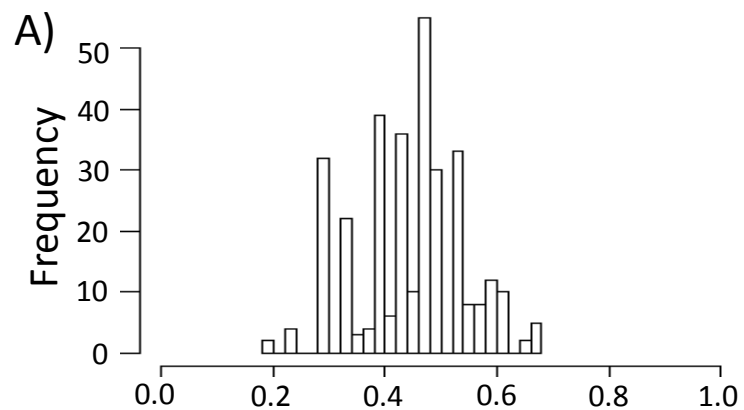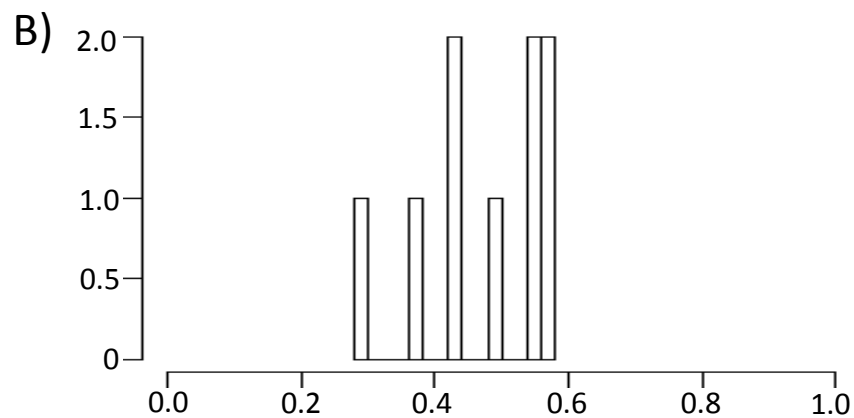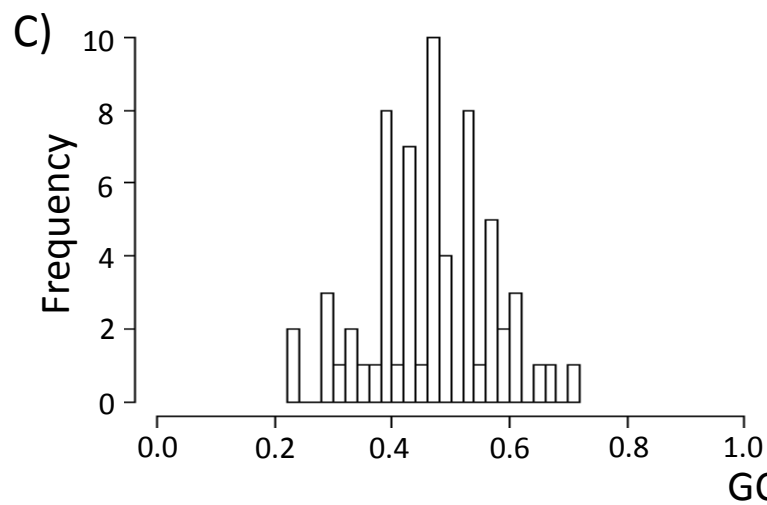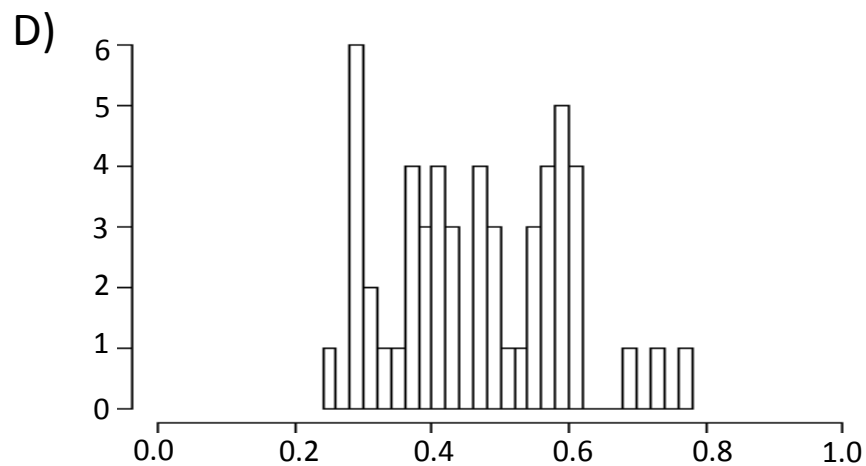

Additional file 6

Supplement: Supplementary file 6 — Additional file 6: Comparison of the GC content distributions for miRNA targets in TPs and FN for Arabidopsis and non-Arabidopsis datasets. (A) and (B) show GC content distribution for the TP and FN miRNAs in Arabidopsis dataset respectively, while (C) and (D) are the plots of GC content distributions for TP and FN datasets in non-Arabidopsis dataset respectively. (PDF 99 KB) [file 12864_2014_6052_MOESM6_ESM.pdf]

Arabidopsis

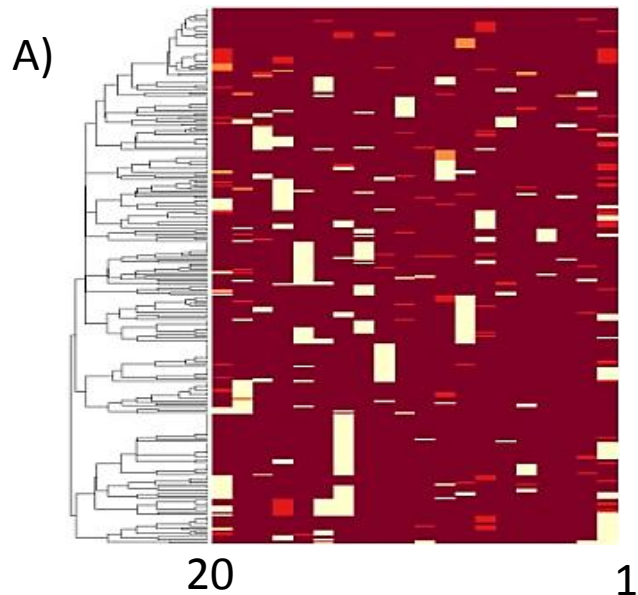

Non-Arabidopsis

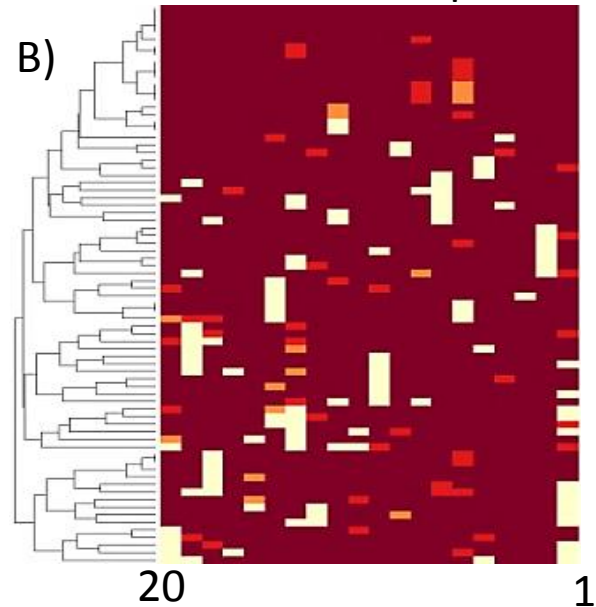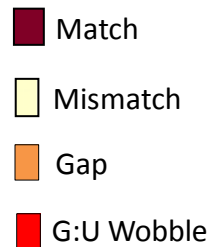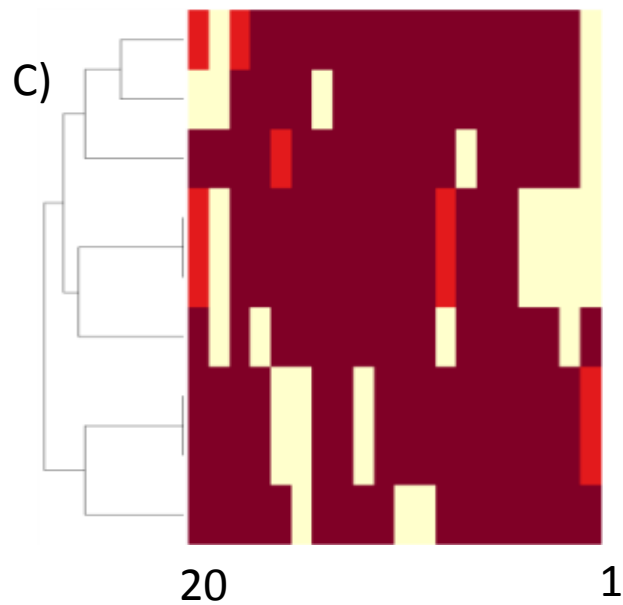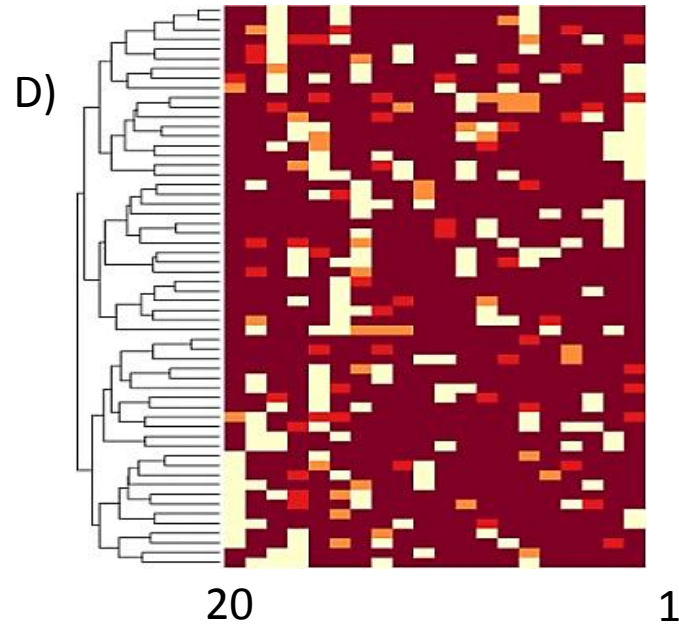

Supplement: Supplementary file 7 — Additional file 7: Heatmaps representing the TP (A and B) and FN (C and D) miRNA-mRNA interactions in Arabidopsis and non-Arabidopsis datasets respectively. (PDF 165 KB) [file 12864_2014_6052_MOESM7_ESM.pdf]
